# Avatrombopag switching from other thrombopoietic agents in severe and persistent cancer treatment-induced thrombocytopenia: a multicentre real-world study

**DOI:** 10.3389/fmed.2026.1898479

**Published:** 2026-07-22

**Authors:** Ye Zhang, Yanan Liu, Junjie Xu, Qingyang Zhang, Xiaojing Zhu, Jingwei Peng, Gege Feng, Jun Peng, Ming Hou, Linlin Shao, Yan Shi

**Affiliations:** 1 Department of Hematology, Qilu Hospital of Shandong University, Jinan, Shandong, China; 2 Department of Gynecological Oncology, Shandong Cancer Hospital and Institute, Shandong First Medical University and Shandong Academy of Medical Sciences, Jinan, Shandong, China; 3 Shandong Key Laboratory of Hematological Diseases and Immune Microenvironment, Jinan, Shandong, China; 4 Shandong Provincial Clinical Research Center for Hematological Diseases, Jinan, Shandong, China; 5 State Key Laboratory of Discovery and Utilization of Functional Components in Traditional Chinese Medicine, Shandong University, Jinan, Shandong, China; 6 Department of Hematology, The First Affiliated Hospital of Shandong First Medical University & Shandong Provincial Qianfoshan Hospital, Jinan, Shandong, China

**Keywords:** avatrombopag, baseline bleeding, baseline hemoglobin level, cancer treatment-induced thrombocytopenia (CTIT), platelet response, switching therapy

## Abstract

**Background:**

Severe and persistent cancer treatment-induced thrombocytopenia (CTIT) remains a major obstacle to continuation of antineoplastic therapy and faces an increased risk of bleeding. Standard therapy has yet to be established in this setting.

**Objectives:**

This study aimed to evaluate the efficacy of avatrombopag switching from other thrombopoietic agents and baseline factors associated with non-response or early response in severe and persistent CTIT.

**Methods:**

In this multicentre real-world cohort study, we included 78 patients with solid tumors or non-myeloid hematologic malignancies who showed no response or loss of response to prior thrombopoietic agents and had grade 4 thrombocytopenia for at least 4 weeks. Platelet response and relevant influencing factors, transfusion dependence, interruptions to antineoplastic therapy, and safety were assessed.

**Results:**

Median baseline platelet count was 10 × 10^9^/L and increased to 102 × 10^9^/L after switching to avatrombopag (*p* < 0.001). Platelet response rates increased over time, from 28.2% at week 4 to 43.6% at week 8 and 52.6% at week 12. The overall difference across these time points was significant (*p* < 0.001). After switching therapy, platelet transfusion dependence decreased significantly from 69.2% to 19.2% (*p* < 0.001), along with interruptions to antineoplastic therapy decreased from 88.5 to 50.0% (*p* < 0.001). The median time to platelet response was 11.7 weeks. Hemoglobin>123 g/L at baseline might be associated with early platelet response. Major bleeding at baseline might independently predicted poor platelet response across different time points. No grade ≥3 adverse events were observed.

**Conclusion:**

Switching to avatrombopag was associated with platelet recovery, reduced transfusion dependence, fewer interruptions to antineoplastic therapy, and an acceptable safety profile for severe and persistent CTIT.

## 1 Introduction

Cancer treatment–induced thrombocytopenia (CTIT) is among the most common hematologic toxicities of systemic antineoplastic therapy in both solid tumors and hematologic malignancies and is generally defined as a peripheral platelet count below 100 × 10^9^/L (1, 2). Its main pathogenesis lies in the impairment of bone marrow hematopoietic function caused by radiotherapy, chemotherapy, immunotherapy and targeted therapy (3–7). CTIT can present as either transient nadir or persistent thrombocytopenia. In most patients with transient nadir thrombocytopenia, platelet counts can recover spontaneously, thrombopoietic agents are only indicated when bleeding events occur or extremely low platelet counts are observed (8). In contrast, persistent CTIT is characterized by failure of platelet recovery beyond the expected period of myelosuppression. This may result in reduced relative dose intensity, treatment delay, or premature discontinuation of antineoplastic therapy, necessitating thrombopoietic supportive care (9). The degree of thrombocytopenia is categorized grades 1 to 4 and the reported incidences of grade 3 or 4 thrombocytopenia range from 1.2 to 28.7%, depending on treatment modality (2, 10–12). Grade 4 thrombocytopenia (platelet count<25 × 10^9^/L) substantially increases bleeding risk and is particularly challenging when this clinical issue persists beyond the expected period of treatment-related marrow suppression. In routine clinical practice, severe and persistent thrombocytopenia is not merely a laboratory abnormality. It often leads to repeated platelet transfusions, unplanned hospital visits, postponement, dose reduction or premature termination of antineoplastic therapy, and increased concern regarding bleeding during subsequent treatment cycles. These consequences are especially relevant for patients who still require ongoing antineoplastic treatment but have already failed to recover platelet counts despite conventional thrombopoietic support (1, 13, 14). Therefore, studies in this setting should evaluate not only platelet count recovery, but also clinically meaningful outcomes such as transfusion dependence, interruptions to antineoplastic therapy, time to platelet response, and safety.

Recombinant human interleukin-11 and recombinant human thrombopoietin (rhTPO) have improved the management of CTIT, yet a subset of patients still develops into severe and persistent thrombocytopenia. Thrombopoietin receptor agonists (TPO-RAs) activate the thrombopoietin receptor, promote the proliferation and maturation of megakaryocytes, and enhance the production of platelet (15, 16). Their successful applications in immune thrombocytopenia, aplastic anemia and periprocedural thrombocytopenia in chronic liver disease have prompted growing interest in their potential clinical value for CTIT (17). However, no TPO-RA has been approved for CTIT globally (13). Administration of romiplostim effectively improved platelet counts, reduced the incidence of platelet transfusions, as well as decreased the need for modifications of chemotherapy dose in patients with chemotherapy-induced thrombocytopenia (18, 19).

In a clinical study of patients with solid tumors who encountered a first episode of grade 3 or 4 thrombocytopenia after chemotherapy, avatrombopag significantly increased the platelet nadir (8). In patients with grade 2 or higher thrombocytopenia after the most recent chemotherapy cycle, avatrombopag shortened the duration of grade 3/4 thrombocytopenia and reduced requirements for platelet transfusion (20). In addition, among CTIT patients with inadequate response to rhTPO or eltrombopag, switching to avatrombopag has been associated with subsequent platelet recovery and transfusion independence, especially for patients with hemoglobin (Hb) >90 g/L at baseline (21). These observations suggest that avatrombopag may increase platelet counts in chemotherapy-induced thrombocytopenia. However, evidence remains limited for patients with severe and persistent CTIT who have no response to one or more prior thrombopoietic agents. The National Comprehensive Cancer Network Hematopoietic Growth Factors Guidelines, Version 3.2026, continue to prioritize enrollment in TPO-RA clinical trials (22), underscoring the lack of established standard therapy in this setting.

In this study, we report a multicenter real-world cohort of patients with solid tumors or non-myeloid hematologic malignancies who present with persistent thrombocytopenia after antineoplastic therapy. Previous treatment with one or more thrombopoietic agents was unsuccessful and grade 4 thrombocytopenia persists for at least 4 weeks before switching to avatrombopag treatment. This cohort represents a clinically perplexing group with limited evidence to guide subsequent treatment. We aim to report real-world evidence on the efficacy of avatrombopag as switching therapy by assessing platelet response at prespecified time points and overall response during treatment. We also aim to examine changes in platelet transfusion dependence and interruptions to antineoplastic therapy, characterize the time to platelet response, explore baseline factors associated with early platelet response and non-response, and report the safety profile during avatrombopag administration.

## 2 Materials and methods

### 2.1 Study population

This was a multicenter retrospective study conducted at two participating medical centers, namely Qilu Hospital of Shandong University and Shandong Cancer Hospital and Institute Affiliated to Shandong First Medical University. Eligible study subjects were retrospectively identified through a review of medical records at each institution. Patients were screened from those who developed severe and persistent thrombocytopenia after receiving antineoplastic therapy for solid tumors or non-myeloid hematologic malignancies between January 2021 and June 2024. Relevant demographic and clinical data, including primary disease characteristics, treatment history, laboratory findings, and follow-up information, were collected from the electronic medical record systems and communication systems of the two centers for subsequent analysis. The study was conducted in accordance with the ethical principles of the Declaration of Helsinki, as revised in 2013. The study protocol was reviewed and approved by the Ethics Committee of the leading center, Qilu Hospital of Shandong University (Approval Number: QLCR20260276). Ethical approval was mutually recognized between different participating centers.

Inclusion criteria included: (a) Adults aged 18 years or older. (b) Patients showed no response or lost response to previous treatment with rhTPO and/or TPO-RAs (eltrombopag / hetrombopag / romiplostim). Nonresponse to rhTPO was defined as refractory to rhTPO 300 U/(kg^.^d) for at least 14 days. Nonresponse to eltrombopag was defined as refractory to eltrombopag 75 mg QD for at least 30 days. Nonresponse to hetrombopag was defined as refractory to hetrombopag 7.5 mg QD for at least 30 days. Nonresponse to romiplostim was defined as refractory to romiplostim 10ug/kg QW for at least 4 weeks. Loss of response to rhTPO and/or TPO-RAs was defined as initial treatment efficacy followed by a progressive or abrupt decline in platelet count and recurrence of bleeding, despite dose escalation to the maximum dose. The baseline platelet counts ranged from 0 to 99 × 10^9^/L prior to previous treatment with rhTPO and/or TPO-RAs (eltrombopag / hetrombopag / romiplostim). If the platelet count before previous treatment was less than 25 × 10^9^/L, the cutoff for nonresponse/loss of response was less than 50 × 10^9^/L. If the baseline platelet count was 25 to 99 × 10^9^/L, the cutoff for nonresponse/loss of response was less than 75 × 10^9^/L or with a rise of ≤25 × 10^9^/L from baseline. (c) Patients who had suffered from grade 4 thrombocytopenia persisting for at least 4 weeks before switching therapy. Grade 4 thrombocytopenia was defined as peripheral blood platelet count <25 × 10^9^/L according to common terminology criteria for adverse events (CTCAE) version 5.0 (23). Exclusion criteria were: (a) Another thrombopoietic agent was initiated during avatrombopag treatment. (b) Patients had received any investigational therapy within 30 days before switching to avatrombopag. (c) Thrombocytopenia was attributable to causes other than CTIT. Avatrombopag was initiated at 40 mg once daily. The dosage and frequency were adjusted according to platelet count and bleeding status. The overall goal of dose modifications was to maintain platelet count ≥50 × 10^9^/L and ≤150 × 10^9^/L. The dose titration was performed according to the following criterion: (a) dose de-escalation considered if platelet count ≥100 × 10^9^/L for at least 2 weeks; (b) de-escalate immediately if platelet count ≥250 × 10^9^/L; (c) escalate if platelet count <50 × 10^9^/L for at least 2 weeks. The maximum maintenance dose of avatrombopag was 60 mg once daily. However, some patients especially who did not require further antineoplastic therapy maintained stable platelet count ≥50 × 10^9^/L after discontinuation of avatrombopag. The baseline date was defined as the date of avatrombopag initiation. Baseline clinical and laboratory variables were collected using the most recent available records before avatrombopag initiation. Treatment was planned for at least 4 weeks unless platelet recovery occurred earlier. Follow-up continued from avatrombopag initiation until the last documented clinical visit, death, or loss to follow-up, whichever occurred first. Because this was a retrospective study based on routine clinical records, treatment decisions, dose adjustments, platelet transfusions, and antineoplastic therapy modifications were made by the treating physicians according to clinical practice.

### 2.2 Data collection

All data were extracted from the hospital’s Electronic Medical Record, Picture Archiving and Communication System. Two investigators (Zhang and Peng) independently extracted data and performed cross verification; any discrepancies were adjudicated by a third reviewer (GF). Collected variables encompassed: sex, age, tumor type and stage, status of liver and bone marrow malignant infiltration, history of antineoplastic treatment (treatment modality, classification of antineoplastic agents and number of treatment cycles), duration of grade 4 thrombocytopenia, degree of bone marrow hyperplasia, megakaryocytic hematopoiesis, number of prior thrombopoietic agents, peripheral absolute neutrophil count, platelet count, hemoglobin (Hb) level, avatrombopag dose, oral duration of avatrombopag, history of platelet transfusion, bleeding severity, and adverse events. Bone marrow aspiration and biopsy were performed at baseline, within 1 month before avatrombopag commencement. In routine clinical practice, patients were followed up for at least 12 weeks. The median follow-up time was 28 weeks (range, 12.0–148.0).

### 2.3 Outcome measures

Platelet response was defined as achievement of a median platelet count of at least 50 × 10^9^/L during avatrombopag treatment or within 7 days after treatment completion, together with at least a 2-fold increase from baseline and absence of bleeding. Complete platelet response was defined as achievement of a platelet count of at least 100 × 10^9^/L. Response was assessed at week 4, week 8, week 12, the time before drug discontinuation or the last follow-up for patients receiving continuous avatrombopag medication. For patients who required rescue therapy, platelet count values obtained within 1 week after rescue treatment were excluded from efficacy assessment. Rescue therapy was defined as platelet transfusion. The primary endpoint was platelet response after switching to avatrombopag, including response at prespecified time points and baseline factors associated with non-response, early response. Secondary endpoints included complete platelet response, platelet transfusion dependence, interruptions and reinitiation of antineoplastic therapy, time to the first documented platelet response, thrombocytosis, thrombotic events, and other adverse events. Platelet transfusion dependence was defined as the requirement for platelet transfusion because of thrombocytopenia or bleeding risk. Interruption to antineoplastic therapy was defined as temporary discontinuation or inability to proceed with planned antineoplastic treatment because of thrombocytopenia. Time to platelet response was calculated from avatrombopag initiation to the first date on which platelet response criteria were met. Thrombocytosis was defined as platelet count >400 × 10^9^/L. Adverse events after switching to avatrombopag were evaluated through review of medical records. Thrombotic events included objectively documented arterial or venous thrombosis during follow-up. Bleeding severity was defined according to definitions by the International Society on Thrombosis and Haemostasis and assessed according to clinical records (24, 25).

### 2.4 Statistical method

Quantitative variables were summarized descriptively. Continuous variables were primarily presented as medians and ranges in order to provide a robust summary of the central tendency and dispersion of the observed data, and then were compared using the paired *t*-test or Wilcoxon signed-rank test for comparisons of paired continuous variables measured at different time points. Categorical variables are presented as counts and percentages. For comparisons of repeated paired categorical data across different time points, Cochran’s Q test was used when more than two related proportions were compared. When only two paired categorical measurements were involved, McNemar’s test was applied. Factors associated with non-response or early response to avatrombopag were first explored using univariable logistic regression. Variables with statistical significance in univariable analyses were entered into the multivariable logistic regression model to further evaluate their independent associations with treatment non-response or early response. Because of the retrospective study design and the limited sample size, the multivariable analyses were considered exploratory in nature. These models were primarily intended to identify potentially clinically relevant associations and hypothesis-generating signals rather than to establish definitive causal relationships. Therefore, the results of regression analyses were interpreted with appropriate caution, particularly in the context of potential residual confounding, limited statistical power, and the inherent constraints of observational data. Time to the first documented platelet response was estimated using the Kaplan–Meier method. This time-to-event approach was used to estimate the cumulative probability of achieving an initial platelet response over time and to account for differences in follow-up duration among patients. Patients who did not achieve a platelet response during the observation period were censored at the time of their last available assessment. Kaplan–Meier estimates were used to describe the temporal pattern of response occurrence in the study cohort. All statistical analyses were performed using IBM SPSS version 27. Visualization was created with IBM SPSS version 27 and final figure assembly was performed using Adobe Illustrator 2026. *p* < 0.05 was considered statistically significant. For logistic regression analyses, non-response was used as the outcome variable; therefore, an odds ratio greater than 1 indicated a higher likelihood of non-response, whereas an odds ratio less than 1 indicated a lower likelihood of platelet response. Because of the retrospective design and limited sample size, multivariable models were considered exploratory and were used to identify clinically relevant associations rather than to establish causality.

## 3 Results

### 3.1 Basic characteristics of patients

A total of 82 adult patients with severe and persistent CTIT who were switched to avatrombopag were included in the study. Among them, 78 patients were evaluable for efficacy, including 70 patients with solid tumors and 8 with hematologic malignancies. Four patients were excluded from the efficacy analysis, including 1 patient who discontinued avatrombopag shortly after treatment initiation and three patients whose follow-up ended prematurely before efficacy could be adequately assessed. Before avatrombopag initiation, 67 patients were refractory to previous rhTPO and/or TPO-RAs, four lost responses, and seven exhibited loss of response to one agent while demonstrating primary nonresponse to alternative agents. Sixty nine patients required ongoing antineoplastic therapy as clinically indicated by the status of the underlying malignancy, highlighting the urgency of platelet recovery. Baseline characteristics of the efficacy-evaluable population are summarized in Table 1. The median age of the cohort was 59 years (range, 22–81), and 41 patients (52.6%) were female. Colorectal cancer, lung cancer, gynecologic malignancies, breast cancer, and hepatobiliary malignancies were the most common tumor types, together accounting for approximately two-thirds of all cases. The median number of different antineoplastic therapy modalities previously administered was 2 (range, 1–4), and the median cumulative number of antineoplastic treatment cycles was 6 (range, 1–31). At baseline, the median platelet count was 10 × 10^9^/L (range, 0–25 × 10^9^/L), reflecting the severity of thrombocytopenia in this cohort. The median interval from the onset of grade 4 thrombocytopenia to avatrombopag initiation was 7.8 weeks (range, 4.0–96.0). Notably, 37 patients (47.4%) had previously received 2 or more thrombopoietic agents before switching to avatrombopag, suggesting that nearly half of the cohort had already undergone multiple supportive treatment attempts. After switching to avatrombopag, the median treatment duration was 25.7 weeks (range, 0.7–148.0).

**Table 1:** Baseline characteristics in patients with solid tumors and hematologic malignancies before switching to avatrombopag.

Characteristic	Overall (*n* = 78)
Age (years), median (range)	59 (22–81)
Female sex, *n* (%)	41 (52.6)
AJCC^a^ stage, *n* (%)	
I	7 (9.0)
II	10 (12.8)
III	23 (29.5)
IV	34 (43.6)
Not AJCC staged^b^	5 (5.1)
Tumor type, *n* (%)	
Breast	8 (10.3)
Colorectal	11 (14.1)
Gastroesophageal	7 (9.0)
Genitourinary	5 (6.4)
Gynecologic	13 (16.7)
Hepatobiliary	8 (10.3)
Lung	13 (16.7)
Lymphoid malignancies	7 (9.0)
Myeloma	1 (1.3)
Neuroendocrine	1 (1.3)
Melanoma	2 (2.6)
Thymic	2 (2.6)
Numbers of types of antineoplastic therapies, median (range)	2 (1–4)
Total cycles of antineoplastic therapies, median (range)	6 (1–31)
Baseline platelet count, ×10^9^/L, median (range)	10 (0–25)
Duration of severe CTIT prior to avatrombopag, weeks, median (range)	7.8 (4.0–96.0)
No. of previous thrombopoietic treatments, *n* (%)	
rhTPO	33 (42.3)
ELT	3 (3.8)
HET	5 (6.4)
rhTPO, ELT	10 (12.8)
rhTPO, HET	20 (25.6)
rhTPO, ROMI	2 (2.6)
ELT, HET	2 (2.6)
rhTPO, ELT, HET	2 (2.6)
rhTPO, ELT, ROMI	1 (1.3)
No. of patients receiving each thrombopoietic agent, *n* (%)	
rhTPO	68 (87.2)
ELT	18 (23.1)
HET	29 (37.2)
ROMI	3 (3.8)
Duration of avatrombopag treatment (weeks), median (range)	25.7 (0.7–148.0)

Data are presented as median (range) or *n* (%).

a AJCC staging was used for solid tumors, and the Lugano modification of Ann Arbor staging was used for lymphomas.

b Includes one patient with myeloma.

AJCC, American Joint Committee on Cancer; CTIT, cancer treatment–induced thrombocytopenia; rhTPO, recombinant human thrombopoietin; ELT, eltrombopag; HET, hetrombopag; ROMI, romiplostim.

### 3.2 Outcomes after switching to avatrombopag in patients with solid tumors and hematologic malignancies

Efficacy outcomes after switching to avatrombopag are summarized in Table 2. The median platelet count after switching was 102 × 10^9^/L (range, 6–461 × 10^9^/L), which was significantly higher than baseline (*p* < 0.001). Platelet response rates increased over time, from 28.2% at week 4 to 43.6% at week 8 and 52.6% at week 12. The overall difference across these time points was significant (Cochran’s Q = 29.16, df = 2, *p* < 0.001). Pairwise comparisons showed significant increases in response rate between weeks 4 and 8 (*p* < 0.001), between weeks 4 and 12 (*p* < 0.001), as well as between weeks 8 and 12 (*p* = 0.02). Over the entire treatment period, the overall platelet response rate was 70.5%, which remained significantly higher than the response rate observed at week 12 alone (*p* = 0.02). A complete platelet response, defined as platelet count ≥100 × 10^9^/L, was achieved in 40 of 78 patients (51.2%). At the time of the first platelet response, 38.1% of responders were receiving avatrombopag 60 mg/day and 61.8% were receiving 40 mg/day. Clinically relevant supportive outcomes are shown in Table 3. After switching to avatrombopag, platelet transfusion dependence decreased significantly from 69.2 to 19.2% (*p* < 0.001). Similarly, the proportion of patients with interruptions to antineoplastic therapy decreased from 88.5 to 50.0% (*p* < 0.001). Collectively, these findings indicate that switching to avatrombopag was associated with improved platelet recovery, increasing response rates over time, reduced transfusion requirements, and fewer interruptions to antineoplastic treatment.

**Table 2:** Outcomes after switching to avatrombopag in patients with solid tumors and hematologic malignancies.

Characteristic	Overall (*n* = 78)
platelet count after switching, ×10^9^/L, median (range)	102 (6–461)
Platelet response at specified time points, *n* (%)	
Response at week 4	22 (28.2)
Response at week 8	34 (43.6)
Response at week 12	41 (52.6)
Platelet response on avatrombopag treatment, *n* (%)	
Response	55 (70.5)
Complete response	40 (51.2)
No response	23 (29.5)
Daily avatrombopag dose at first platelet response, *n* (%)	
40 mg/day	34 (61.8)
60 mg/day	21 (38.1)

**Table 3:** Comparison of platelet transfusion dependence and interruptions to antineoplastic therapy before and after switching to avatrombopag.

Characteristic	Before switching (*n* = 78)	After switching (*n* = 78)	*p* value
Platelet transfusion dependence, *n* (%)	54 (69.2)	15 (19.2)	<0.001
Interruptions to antineoplastic therapy, *n* (%)	69 (88.5)	39 (50.0)	<0.001

**Figure 1:**
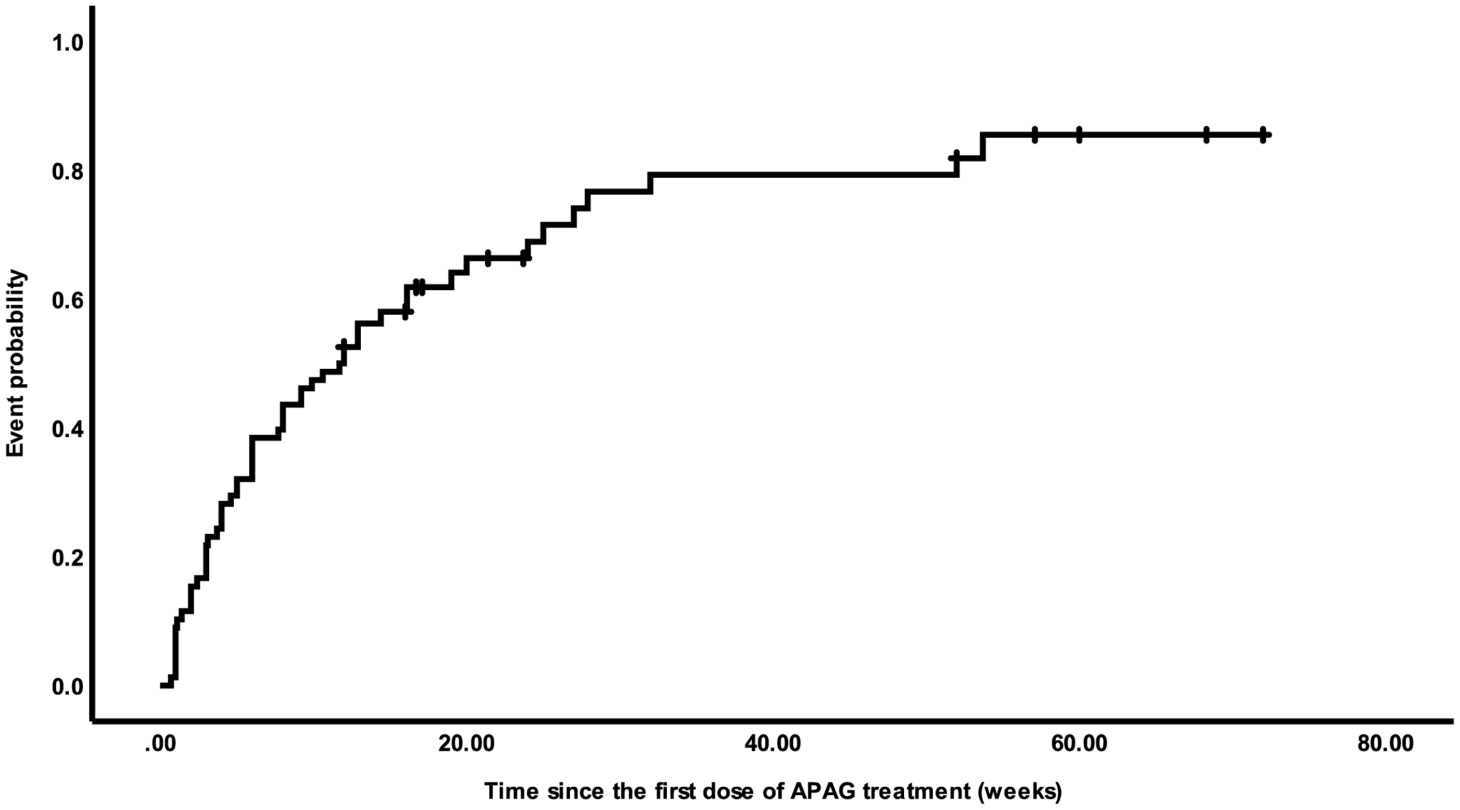
Kaplan–Meier curve of time to platelet response on APAG therapy. The *y*-axis represents the cumulative probability of achieving a platelet response, and the *x*-axis represents time since the first dose of APAG (in weeks). The median time to response was 11.7 weeks (95% CI: 7.4–16.0). Censored observations are indicated by tick marks on the curve.

Figure 1 shows the Kaplan–Meier analysis of time to platelet response among 78 patients treated with avatrombopag. The median time to response was 11.7 weeks (95% CI, 7.4–16.0), indicating that half of the patients who achieved a response did so within approximately 12 weeks of treatment initiation. The restricted mean time to response was 22.0 weeks (95% CI, 15.7–28.3), providing an estimate of the average time to response. The cumulative probability of response increased steadily over the first 24 weeks of treatment, with the majority of responses occurring within the first 16 weeks. After this period, additional responses continued to occur, although at a slower rate. Patients who did not achieve a platelet response by the end of follow-up were censored at their last available assessment. These results indicate that avatrombopag treatment was associated with a gradual increase in platelet response over time, with most responses observed during the early phase of therapy.

### 3.3 Predictors of avatrombopag non-response

Baseline factors associated with platelet response after switching to avatrombopag are summarized in Table 4. In logistic regression analyses, non-response was used as the dependent variable; therefore, odds ratios (ORs) < 1 indicate a lower likelihood of non-response and a higher probability of platelet response. At week 4, baseline Hb > 123 g/L (OR, 0.20; 95% CI, 0.06–0.69; *p* = 0.01) and active bone marrow hyperplasia (OR, 0.27; 95% CI, 0.09–0.79; *p* = 0.02) were associated with platelet response in univariable analysis. In multivariable analysis, Hb > 123 g/L remained independently associated with platelet response at week 4 (OR, 0.22; 95% CI, 0.05–0.96; *p* = 0.04). At week 8, Hb > 123 g/L (OR, 0.25; 95% CI, 0.07–0.90; *p* = 0.03), major bleeding (OR, 4.52; 95% CI, 1.54–13.29; *p* = 0.006), active bone marrow hyperplasia (OR, 0.21; 95% CI, 0.08–0.59; *p* = 0.003), and megakaryocytopenia (OR, 0.28; 95% CI, 0.10–0.75; *p* = 0.01) were associated with response in univariable analysis, whereas multivariable analysis identified major bleeding as an independent predictor of non-response (OR, 6.19; 95% CI, 1.46–26.30; *p* = 0.01). At week 12, major bleeding (OR, 4.22; 95% CI, 1.47–12.07; *p* = 0.007) and active bone marrow hyperplasia (OR, 0.27; 95% CI, 0.10–0.76; *p* = 0.01) were associated with response in univariable analysis; major bleeding remained independently associated with non-response in multivariable analysis (OR, 5.67; 95% CI, 1.43–22.38; *p* = 0.01). During the overall avatrombopag treatment period, age (OR, 1.06; 95% CI, 1.01–1.11; *p* = 0.02), oral duration of avatrombopag (OR, 0.98; 95% CI, 0.95–0.99; *p* = 0.03), major bleeding (OR, 4.16; 95% CI, 1.30–13.33; *p* = 0.012), active bone marrow hyperplasia (OR, 0.27; 95% CI, 0.08–0.93; *p* = 0.04), and prior anthracycline exposure (OR, 0.13; 95% CI, 0.03–0.52; *p* = 0.004) were associated with response in univariable analysis. In forward stepwise multivariable analysis, major bleeding remained an independent predictor of non-response (OR, 6.41; 95% CI, 1.50–27.03; *p* = 0.01), while longer avatrombopag exposure remained significantly associated with response (OR, 0.97; 95% CI, 0.95–1.00; *p* = 0.047). However, the upper bound of the 95% CI reached the line of no effect, suggesting the result is marginally significant and requires cautious interpretation, given the limited sample size. Overall, baseline major bleeding was the most consistent factor associated with a reduced likelihood of platelet response after switching to avatrombopag. Sex, tumor stage, number of treatment cycles, liver and bone marrow malignant infiltration, baseline platelet count, duration of grade 4 thrombocytopenia, history of platelet transfusion, number of prior thrombopoietic agents and neutropenia were not associated with response to avatrombopag. Univariate analysis revealed that baseline Hb level and bleeding severity were significantly correlated with response to avatrombopag treatment. Such correlations remained statistically significant in the multivariate model adjusted for confounding factors. Given the limited sample size of this study, the 95% CIs were relatively wide, which compromised the stability of effect estimation. Further verification in larger cohort studies is required.

**Table 4:** Baseline factors associated with platelet response at week 4, week 8, week 12, and on avatrombopag treatment: univariate and multivariable logistic regression analyses.

Baseline parameter	Univariate		Multivariate	
	OR (95% CI)	*p* value	OR (95% CI)	*p* value
Baseline factors associated with platelet response at week 4				
Sex	1.44 (0.53–3.92)	0.47	N/A	N/A
Age	1.01 (0.97–1.05)	0.57	N/A	N/A
Tumor stage	0.98 (0.58–1.66)	0.94	N/A	N/A
Number of treatment cycles	0.92 (0.84–1.00)	0.06	N/A	N/A
Prior anthracycline exposure	0.78 (0.19–3.26)	0.74	N/A	N/A
Liver malignant infiltration	1.27 (0.38–4.25)	0.70	N/A	N/A
Bone marrow malignant infiltration	0.79 (0.08–8.08)	0.85	N/A	N/A
Active bone marrow hyperplasia	0.27 (0.09–0.79)	0.02	0.32 (0.08–1.23)	0.10
Megakaryocytopenia	0.44 (0.15–1.23)	0.12	N/A	N/A
Hb > 123 g/L	0.20 (0.06–0.69)	0.01	0.22 (0.05–0.96)	0.04
Neutropenia	0.56 (0.19–1.66)	0.30	N/A	N/A
Platelet count	1.00 (0.93–1.08)	1.00	N/A	N/A
Major bleeding	0.36 (0.11–1.14)	0.08	N/A	N/A
History of platelet transfusion	0.75 (0.17–3.32)	0.71	N/A	N/A
Duration of grade 4 thrombocytopenia	1.02 (0.96–1.09)	0.52	N/A	N/A
Number of prior thrombopoietic agents	1.44 (0.53–3.92)	0.47	N/A	N/A
Baseline factors associated with platelet response at week 8				
Sex	1.57 (0.63–3.86)	0.33	N/A	N/A
Age	1.03 (0.99–1.07)	0.17	N/A	N/A
Tumor stage	1.14 (0.71–1.83)	0.58	N/A	N/A
Number of treatment cycles	0.99 (0.91–1.07)	0.78	N/A	N/A
Prior anthracycline exposure	0.35 (0.09–1.41)	0.14	N/A	N/A
Liver malignant infiltration	1.07 (0.34–3.33)	0.91	N/A	N/A
Bone marrow malignant infiltration	0.38 (0.04–3.87)	0.42	N/A	N/A
Active bone marrow hyperplasia	0.21 (0.08–0.59)	0.003	0.09 (0.01–1.33)	0.08
Megakaryocytopenia	0.28 (0.10–0.75)	0.01	2.09 (0.16–27.86)	0.58
Hb > 123 g/L	0.25 (0.07–0.90)	0.03	0.13 (0.01–1.52)	0.10
Neutropenia	0.38 (0.14–1.04)	0.06	N/A	N/A
Platelet count	0.99 (0.93–1.06)	0.71	N/A	N/A
Major bleeding	4.52 (1.54–13.29)	0.006	6.19 (1.46–26.30)	0.01
History of platelet transfusion	0.59 (0.15–2.40)	0.46	N/A	N/A
Duration of grade 4 thrombocytopenia	0.99 (0.95–1.03)	0.67	N/A	N/A
Number of prior thrombopoietic agents	1.27 (0.52–3.11)	0.61	N/A	N/A
Baseline factors associated with platelet response at week 12				
Sex	1.10 (0.45–2.67)	0.84	N/A	N/A
Age	1.02 (0.98–1.06)	0.28	N/A	N/A
Tumor stage	1.02 (0.64–1.62)	0.95	N/A	N/A
Number of treatment cycles	0.98 (0.90–1.07)	0.61	N/A	N/A
Prior anthracycline exposure	0.36 (0.10–1.35)	0.13	N/A	N/A
Liver malignant infiltration	0.94 (0.30–2.91)	0.91	N/A	N/A
Bone marrow malignant infiltration	0.26 (0.03–2.61)	0.25	N/A	N/A
Active bone marrow hyperplasia	0.27 (0.10–0.76)	0.01	N/A	N/A
Megakaryocytopenia	0.37 (0.14–0.99)	0.05	N/A	N/A
Hb > 123 g/L	0.41 (0.12–1.48)	0.17	N/A	N/A
Neutropenia	0.47 (0.18–1.27)	0.14	N/A	N/A
Platelet count	0.99 (0.92–1.05)	0.69	N/A	N/A
Major bleeding	4.22 (1.47–12.07)	0.007	5.67 (1.43–22.38)	0.01
History of platelet transfusion	0.53 (0.12–2.31)	0.40	N/A	N/A
Duration of grade 4 thrombocytopenia	1.00 (0.96–1.04)	0.90	N/A	N/A
Number of prior thrombopoietic agents	1.35 (0.55–3.29)	0.51	N/A	N/A
Baseline factors associated with platelet response on avatrombopag treatment				
Sex	1.02 (0.39–2.71)	0.96	N/A	N/A
Age	1.06 (1.01–1.11)	0.02	N/A	N/A
Tumor stage	1.00 (0.60–1.67)	0.99	N/A	N/A
Number of treatment cycles	1.01 (0.92–1.11)	0.87	N/A	N/A
Prior anthracycline exposure	0.13 (0.03–0.52)	0.004	N/A	N/A
Liver malignant infiltration	0.59 (0.18–1.92)	0.38	N/A	N/A
Bone marrow malignant infiltration	0.11 (0.01–1.08)	0.06	N/A	N/A
Active bone marrow hyperplasia	0.27 (0.08–0.93)	0.04	N/A	N/A
Megakaryocytopenia	0.46 (0.15–1.40)	0.17	N/A	N/A
Hb > 123 g/L	0.39 (0.08–1.94)	0.25	N/A	N/A
Neutropenia	0.39 (0.12–1.23)	0.11	N/A	N/A
Platelet count	0.97 (0.90–1.04)	0.40	N/A	N/A
Major bleeding	4.16 (1.30–13.33)	0.02	6.41 (1.50–27.03)	0.01
History of platelet transfusion	0.71 (0.13–3.72)	0.68	N/A	N/A
Duration of grade 4 thrombocytopenia	1.01 (0.97–1.05)	0.58	N/A	N/A
Number of prior thrombopoietic agents	1.31 (0.49–3.47)	0.59	N/A	N/A
Oral duration of avatrombopag, weeks	0.98 (0.95–0.99)	0.03	0.97 (0.95–1.00)	0.047

Hb, hemoglobin; N/A, not included in the multivariable model.

### 3.4 Safety

Among the 78 patients evaluated during follow-up, thrombocytosis was observed in only 1 patient (1.3%). The highest platelet count recorded in this patient was 461 × 10^9^/L. Importantly, no thrombotic event occurred in association with this episode of thrombocytosis, and the platelet count returned rapidly to the normal range after discontinuation of avatrombopag. Overall, 2 thrombotic events were documented during the follow-up period, including 1 case of abdominal aortic thrombosis and 1 case of cerebral arterial thrombosis, corresponding to an overall thrombotic event rate of 2.6%. No grade 3 or higher adverse events were observed throughout the study. In addition, no newly diagnosed malignancies were identified, and no deaths were attributed to bleeding, thrombosis, or thromboembolism.

## 4 Discussion

In recent years, the emergence of thrombopoietic agents such as eltrombopag, romiplostim, hetrombopag and avatrombopag has aroused great interest among hematology scholars in the pharmacotherapy of CTIT (1, 26, 27). Current guidelines still prioritize enrollment in TPO-RA clinical trials for CTIT, highlighting the lack of a standard treatment (22). In this multicenter real-world study, switching to avatrombopag after failure of, or loss of response to, one or more prior thrombopoietic agents was associated with clinically meaningful platelet recovery and acceptable safety. To our knowledge, this is the largest study of avatrombopag switching therapy and the first retrospective real-world analysis focused on severe and persistent CTIT. Prior studies have shown that avatrombopag can improve platelet counts in patients with chemotherapy-induced thrombocytopenia (8, 20). However, most enrolled patients had less severe thrombocytopenia or bleeding, shorter treatment-related platelet nadirs, or a higher likelihood of spontaneous recovery during subsequent chemotherapy cycles. Data specifically addressing avatrombopag switching therapy after failure of prior thrombopoietic agents remain limited to case series and a clinical study with relatively small cohort size (21, 28). Our study extends these data by including a larger cohort, longer follow-up, as well as an analysis of factors associated with non-response and early response. This cohort represents a particularly difficult population: all evaluable patients had grade 4 thrombocytopenia lasting at least 4 weeks before switching, and nearly half had previously received two or more thrombopoietic treatments. This distinction is important because severe and persistent CTIT is more likely to reflect limited hematopoietic reserve, cumulative treatment-related marrow injury (29, 30) as well as ongoing tumor burden. Therefore, the response observed in our cohort provides a real-world evidence for a population that is often underrepresented in prospective CTIT studies.

Despite this unfavorable baseline context, 70.5% of patients achieved platelet response on avatrombopag treatment, 51.2% achieved complete response. In parallel, platelet transfusion dependence decreased substantially, and a markedly smaller number of patients experienced interruptions to antineoplastic therapy after switching. Platelet transfusion remains an important rescue strategy as supportive care for severe thrombocytopenia, especially in patients with bleeding or very low platelet counts, but repeated transfusions may contribute to repeated hospital visits, treatment delay, and patient burden (31). Platelet transfusion dependence may also be associated with limited durability of platelet increments in heavily treated cancer patients (32). Although this retrospective study cannot separate the effect of avatrombopag from all concurrent clinical factors, the magnitude of change supports the potential value of avatrombopag switching as part of supportive care for severe and persistent CTIT. In patients who still require active antineoplastic treatment, recovery of platelet counts may therefore have consequences beyond platelet transfusion dependence. Decisions to resume antineoplastic therapy may depend on tumor status, performance status, infection, organ function, preference of patients, and judgment of physicians. Nevertheless, the observed reduction in interruptions to antineoplastic therapy suggests that platelet recovery after avatrombopag switching may help restore treatment feasibility in a subset of patients. These findings suggest that avatrombopag switching may provide clinical benefit beyond numerical platelet recovery in patients with severe and persistent CTIT.

The response pattern in our study also deserves consideration. In exploratory association analysis, a baseline Hb > 123 g/L was associated with earlier platelet response. Although the result was statistically significant, the wide confidence interval indicated some uncertainty in the estimation of effect size. The platelet response rate rose gradually with prolonged duration of avatrombopag medication, suggesting that patients with severe and persistent CTIT may require longer exposure of TPO-RA before meeting response criteria. The median time to first documented platelet response was 11.7 weeks, supporting the need for adequate treatment duration before declaring treatment failure, provided that the patient remains clinically stable and does not experience unacceptable adverse events. Previous *in vitro* cellular experiments had demonstrated that avatrombopag promoted megakaryopoiesis at the critical stage of megakaryocyte maturation in a dose-dependent manner. Humanized mouse models further confirmed that oral avatrombopag increased human platelet counts in mice also in a dose-dependent fashion, and the peak plasma concentration of free drug in mice was comparable to that observed in human subjects receiving therapeutic oral doses (33, 34). To a certain extent, decisions of prolonged treatment can be individually provided according to bleeding risk, platelet trajectory, ongoing antineoplastic treatment needs, and thrombotic risk.

In exploratory association analysis, while the estimation of effect size carried some degree of uncertainty, major bleeding at baseline was the most consistent predictor of poor platelet response. It independently predicted non-response at week 8, week 12, and during avatrombopag treatment. This finding may have several explanations. First, baseline major bleeding may identify patients with higher ongoing platelet consumption. Second, it may reflect advanced disease, organ dysfunction, infection, mucosal injury, or other systemic conditions that impair platelet recovery. Third, patients with major bleeding may require more frequent transfusions and treatment interruptions, making platelet response more difficult to assess and sustain. Most prospective trials of TPO-RAs have excluded patients with active bleeding at enrollment, so evidence on this subgroup remains limited. Our data suggest that major bleeding should be considered when estimating the likelihood of response to avatrombopag switching and when discussing expected benefit with patients.

The efficacy of avatrombopag appeared to be unaffected by the number of types of prior thrombopoietic agents, which may be explained in part by pharmacologic differences among thrombopoietic agents. Previous real-world observational studies demonstrated that switching therapy between avatrombopag and other thrombopoietic agents is feasible, suggesting an absence of cross-resistance and an existence of distinct efficacy profiles among different thrombopoietic agents (35, 36). Avatrombopag binds to the transmembrane domain of the thrombopoietin receptor and does not compete with endogenous thrombopoietin or exogenous rhTPO for receptor binding (33, 37–40). Although both avatrombopag and eltrombopag target the histidine residue at position 499 in the transmembrane region of the receptor, avatrombopag has demonstrated greater *in vitro* activity in promoting megakaryocyte differentiation and colony formation from human cord blood CD34(+) cells and has significantly increased the number of human platelets in non-obese diabetic/severe combined immunodeficiency mice with transplanted human hematopoietic stem cells (41). A study of TPO-RAs switching therapy in pediatric ITP patients unresponsive to hetrombopag or eltrombopag demonstrated that avatrombopag had safe and effective potential as a bridging therapy and could reduce bleeding, ITP medications, and rescue therapy (35). These differences may partly indirectly explain why some patients respond to avatrombopag after failure of prior thrombopoietic therapy.

Safety is a central concern when using TPO-RAs in patients with cancer. Cancer itself is associated with an increased risk of arterial and venous thromboembolism, and many patients also have additional risk factors, including advanced age, active malignancy, surgery, chemotherapy, antiangiogenic therapy, immobility, infection, and central venous catheters (42, 43). In our study, avatrombopag was generally well tolerated. Thrombocytosis occurred in only one patient and resolved after avatrombopag discontinuation. No venous thromboembolism was observed. Two arterial thrombotic events were observed, corresponding to an overall thrombotic event rate of 2.6%, and neither event occurred in the setting of thrombocytosis. The overall thrombotic event rate was not higher than that reported in comparable oncology populations (44). These findings suggest acceptable tolerability of avatrombopag in severe and persistent CTIT cohort, but it should not be interpreted as proof of absence of thrombotic risk. Careful monitoring remains necessary, particularly in patients with active cancer and baseline vascular risk factors.

The potential relationship between platelet recovery and tumor progression also requires caution. Platelets play an active role in multiple processes of cancer biology, including evading immune surveillance, adhering to vascular endothelium, undergoing transendothelial migration, as well as establishing and maintaining the microenvironments of primary and metastatic tumors (45, 46). Given the recognized possible links between platelets and tumor progression, the long-term safety of prolonged avatrombopag exposure still requires further study. Our study did not observe new malignancies during follow-up, but it was not designed to evaluate long-term oncologic outcomes. Future studies should assess whether prolonged avatrombopag exposure influences tumor-related outcomes or survival in specific cancer cohorts. Such analyses will require larger cohorts, longer follow-up, and appropriate adjustment for tumor type, stage, treatment modality, and baseline thrombotic risk.

This study has several limitations. The cohort was heterogeneous with respect to tumor type, prior antineoplastic therapy, status of bone marrow hematopoietic function and previous thrombopoietic treatments. Selection bias cannot be excluded. Although this heterogeneity reflects real-world practice, it limits the interpretation about subgroup. This was a small-sample retrospective analysis, especially for hematologic malignancies, and multivariable analyses should be considered exploratory. The wide 95% CIs for baseline variables, such as Hb > 123 g/L and major bleeding, indicated uncertainty in the estimation of effect sizes. Nevertheless, none of the two CIs crossed the line of no effect (OR = 1), and the direction of effects of major bleeding remained largely consistent between univariate and multivariate analyses in all time points, confirming the statistically significant associations with treatment response. The broad CIs were primarily attributable to the limited sample size and insufficient endpoint events. Accordingly, the multivariate analyses in the present study were performed merely for exploratory association analysis. Further validation in large-scale prospective cohort studies is required to precisely quantify the effect sizes.

## 5 Conclusion

Taken together, avatrombopag switching therapy was associated with platelet recovery, reduced transfusion dependence, and improved feasibility of antineoplastic therapy in a real-world cohort of patients with severe and persistent CTIT after failure of one or more prior thrombopoietic agents. Major bleeding at baseline appeared to identify patients with lower likelihood of platelet response. Avatrombopag was generally well tolerated in this cohort, although thrombotic risk and long-term oncologic safety require continued attention. These findings support avatrombopag as a reasonable switching option for selected patients with severe and persistent CTIT and provide a basis for prospective studies in this complicated clinical setting.

## Data Availability

The original contributions presented in the study are included in the article/supplementary material, further inquiries can be directed to the corresponding authors.
